# Potentially avoidable emergency department visits among patients with advanced cancer

**DOI:** 10.3332/ecancer.2025.1984

**Published:** 2025-09-03

**Authors:** Miguel Araujo-Meléndez, Mirza Jacqueline Alcalde-Castro, Andrea De-la-O-Murillo, Thierry Hernandez-Gilsoul, Enrique Soto-Perez-de-Celis, Roberto Gonzalez-Salazar, Yanin Chavarri-Guerra

**Affiliations:** 1 Hospital Lomas y Hospital de la Salud, San Luis Potosí 78218, México; 2 Divisions of Palliative Medicine and Medical Oncology, Department of Medicine, University of Toronto, Toronto M5G 2C1, Canada; 3 Hospital Infantil de México Federico Gómez, Ciudad de México 06720, México; 4 Continuous Institutional Care and Emergency Department, Instituto Nacional de Ciencias Médicas y Nutrición Salvador Zubirán, Ciudad de México 14000, México; 5 Division of Medical Oncology, University of Colorado Anschutz Medical Campus, Aurora, CO 80045, USA; 6 Department of Hematology and Oncology, Instituto Nacional de Ciencias Médicas y Nutrición Salvador Zubirán, Ciudad de México 14000, México

**Keywords:** emergency department visits, advanced cancer, low- and middle-income countries, quality of care, supportive care

## Abstract

**Background:**

Potentially avoidable emergency department (ED) visits are considered an indicator of the quality of cancer care.

**Objective:**

To investigate the causes of ED visits of patients with advanced cancer.

**Methods:**

We included in this analysis the visits to the ED of patients with advanced cancer in a tertiary cancer center in Mexico City, registered the reasons for their visit, and classified them as potentially avoidable or not by three independent observers.

**Results:**

Seventy-seven patients were included, and 69% had at least one visit to the ED. Fifty-seven percent of visits were classified as potentially avoidable. The most common causes of visiting the ED were: pain, gastrointestinal disorders and ascites. Patients with gastrointestinal and genitourinary tumours had a higher frequency of unavoidable ED visits compared to patients with other tumours (43.3% versus 20.7%, *p* 0.03).

**Conclusion:**

A significant proportion of patients with advanced cancer visit the ED and many of these visits were classified as potentially avoidable based on expert judgment and adapted criteria. These findings highlight the need for further research and context-specific strategies, such as care and early palliative integration, to safely reduce unnecessary ED use and enhance quality of life in low- and middle-income settings.

## Introduction

Cancer incidence and mortality have grown considerably, mainly in emerging countries [1]. Likewise, the number of patients with cancer that visit Emergency Departments (EDs) has been increasing due to an increase in the prevalence of patients with cancer due to population aging, [2] and to the increase in survival due to new treatment strategies [3]. However, this has also increased aggressive cancer care near the end of life, leading to frequent ED visits, admission to intensive care units (ICU) and an underuse of palliative care [4]. ‘Aggressive cancer care near the end of life’ or ‘aggressive end-of-life cancer care’ is defined as on or more of the following criteria: new chemotherapy regimen starting less than 30 days before death, the last dose of chemotherapy withing 14 days of death, more than one emergency visit in the last month of life, more than 14 days in hospital in the last month of life, more than one hospital admission in the last moth of life or ICU admission in the last month of life’ [5]. On the other hand, it is important to emphasise the difference with ‘palliative care’ defined as an approach that improves the quality of life of patients and their families who are facing problems associated with life-threatening illness, preventing and relieving suffering through the early identification, correct assessment and treatment of pain and other problems, whether physical, psychosocial or spiritual [6].

Most patients with cancer visit the ED at least once during the course of their illness [7] and it has been reported that up to 22.4% of patients experience at least one potential incident of aggressive cancer care near the end of life [8]. Among the indicators of poor quality care for patients with advanced cancer are making multiple visits to the ED, multiple hospital admissions, admission to the ICU, the use of chemotherapy in the last 14 days of life and in-hospital death [4]. Unfortunately, a significant proportion of ED visits of patients with advanced cancer result from inadequately controlled treatment-related symptoms, such as pain, nausea or dehydration, that are potentially preventable through better outpatient care. Therefore, a decrease in potentially avoidable ED visits is essential to improve the quality of treatment of patients with cancer and to optimise existing resources [9]. Little information exists regarding potentially avoidable ED visits by patients with cancer, particularly in developing countries where the availability of EDs is limited. According to Medicare and Medicaid in the United States, the definition of potentially avoidable ED visits for patients with adverse effects related to chemotherapy includes 10 symptoms that are considered to have well-established evidence for their outpatient treatment and prevention [10] under the concept that these clinical problems can be managed in more accessible and resolutive operating models (for example, outpatient, home and telephone clinics) [11].

Our aim in this study was to explore the clinical characteristics of recently diagnosed patients with advanced solid tumours, the causes for their ED visits and the frequency of potentially avoidable visits.

## Methods

This study was conducted in the ED of the Instituto Nacional de Ciencias Médicas y Nutrición Salvador Zubirán (INCMNSZ), a tertiary hospital for adults in Mexico City with 156 beds and an oncology service. In 2016, the INCMNSZ received 36,687 all-cause ED visits.

We included patients with newly diagnosed advanced solid tumours between October 2015 and March 2016 and followed their visits to the ED for a year. Sociodemographic data such as age, sex, availability of health insurance and primary tumour site were registered. Tumours were classified according to their origin in the lung, breast, gastrointestinal system, genitourinary system and others. To identify potentially avoidable visits, we used adapted criteria from Medicare and Medicaid guidelines [10], which focus on common complications related to cancer treatment. These were complemented by independent review from three board-certified clinical experts in oncology and palliative care. Reviewers classified the following reasons for ED visits as potentially avoidable: anemia, nausea, vomiting, dehydration, neutropenia, diarrhea, pain, pneumonia and fever or sepsis. ED visits not explicitly addressed by the original guidelines were categorised based on expert consensus. Although these criteria were developed in a different health system, their structured format provided a reproducible approach in the absence of validated local tools for our local context.

### Statistical analysis

Interobserver agreement was calculated using the kappa coefficient. All the variables collected were described with descriptive statistics, including measures of central tendency according to their distribution. The chi-square test was used to compare the frequency of visits by site of cancer origin.

### Ethical disclosures

This work was approved by the ethics and research committee of the INCMNSZ. Due to the retrospective design of this study, informed consent was not required. The authors declare that they have not used any type of generative artificial intelligence for the writing of this manuscript, nor for the creation of table captions and/or figure legends.

## Results

During the study period, 77 patients were diagnosed with an advanced solid tumour in our center. The mean age was 63 years (range 19–88) and 47% (*n* = 25) were men. Regarding their educational level, 7% had not attended school and 20% had an educational level below middle school. Forty percent did not have any type of health insurance and approximately a third were unemployed. Fifty-three percent (*n* = 39) had gastrointestinal and 21% (*n* = 16) had genitourinary neoplasms. The remaining 26% of tumours corresponded to lung, breast, head and neck and soft tissue cancers. The sociodemographic and clinical characteristics of patients are shown in Table 1.

With a median follow-up of 414 days (SD 613), 96 ED visits were identified. Of all patients, 49 (63.6%) had at least one ED visit, with a median of 1 ED visit per patient (range 1–5). In descending order, the three main reasons for visiting the ED were gastrointestinal symptoms (21.8%), infections (18.7%) and pain (15.6%), with half occurring in patients who had received chemotherapy. Of all visits, 63.5% were classified as potentially avoidable. Interobserver agreement on the classification of ED visits as potentially avoidable or not was moderate (kappa 0.56), reflecting the inherent clinical complexity of retrospective assessment and underscoring the need for more context-specific tools to evaluate avoidability in patients with advanced cancer. The most common cause for visiting the ED classified as potentially avoidable was pain (24.5%), followed by gastrointestinal disorders (22.9%) and ascites (22.9%). The most common causes of ED visits are shown in Table 2. The total number of unavoidable ED visits was significantly higher in patients with gastrointestinal and genitourinary tumours compared to patients with other tumours (43.3% versus 20.7%, *p* 0.03).

## Discussion

This study analysed patients with newly diagnosed advanced solid tumours treated at a tertiary care hospital in Mexico City over a 12-month period. Our results show that more than two-thirds of these patients visited the ED during their first year after diagnosis, and that over half of these visits were classified as potentially avoidable.

Previous studies have reported similar rates of potentially avoidable ED visits after chemotherapy, [12] with an average of 1.4 visits per patient. In our cohort, a greater demand for emergency care was observed among patients with gastrointestinal and urological tumours, who accounted for two-thirds of the sample. This profile differs from that reported in other series [12, 13] and may reflect our institution’s status as a referral center for these types of tumours. Additionally, it is known that patients with gastrointestinal tumours have a significant burden of symptoms [14].

The percentage of potentially avoidable ED visits in our study was notably higher (60%) than that reported by Delgado-Guay [15] in the United States (US), which was 23% of 200 ED visits. This discrepancy may be attributed to the criteria used to define potentially avoidable ED visits, as certain conditions, such as pneumonia and sepsis, are considered controversial. These terms were included in the Medicare model to extend the criteria for severe neutropenia and fever in patients presenting to the ED within the first 30 days following chemotherapy [10]. However, this definition specifically applies to patients receiving chemotherapy and does not consider the effects of other contemporary therapies, such as immunotherapy.

For the purposes of this study, we classified the following conditions as potentially avoidable: anemia, nausea, vomiting, dehydration, neutropenia, diarrhea, pain, pneumonia and fever or sepsis. ED visits not covered by the original Medicare/Medicaid criteria were categorised based on expert consensus, which may also account for differences in classification. These definitional discrepancies contribute to the heterogeneity of reported avoidable rates in the literature.

While identifying potentially avoidable visits is an important first step, we acknowledge that symptoms such as pain and fever in advanced cancer care may evolve unpredictably. Therefore, labeling a visit as ‘potentially avoidable’ should not be interpreted as a critique of clinical decisions, but rather as an opportunity to optimise care delivery and strengthen outpatient management systems.

It is also important to consider the limited access to outpatient palliative and supportive care clinics in resource-limited countries like Mexico, as this context may further impact the patterns of ED visits. Due to a scarcity of chronic care institutions for patients near the end of life, many complications are often treated in the ED, even though this may not be the most appropriate setting. Therefore, avoiding unnecessary interventions and ED admission is crucial to ensure patient comfort and minimise distress for both patients and their families, particularly at the end of life, regardless of the classification system used. Additionally, unnecessary ED visits and hospital admissions can lead to aggressive interventions and increased cost [16].

We advocate for strengthening outpatient and palliative care services through context-adapted strategies. These may include rapid symptom control clinics, structured telephone triage protocols, early integration of palliative care into oncology follow-up, and basic palliative training for primary care providers. Such initiatives could reduce reliance on emergency services without compromising patient safety.

Our study has some limitations. First, we recognise that the criteria used to classify visit avoidability were originally developed in the US and primarily focused on chemotherapy-related complications. In the absence of validated tools in Latin America, we adapted these guidelines as an initial framework and complemented them with clinical review by experts familiar with local care pathways. The moderate inter-rater agreement observed highlights the complexity of the assessment and the urgent need for the development and validation of context-specific avodability tools for use in low- and middle- income countries. Second, due to the retrospective nature of this study, we were unable to evaluate important variables such as patient distress, quality of life or healthcare costs. These outcomes are essential to fully understanding the impact of avoidable ED visits and should be prioritised in future prospective studies.

Third, we could not account for visits to other institutions outside our center, which may have resulted in underestimation of ED use. Finally, we acknowledge that our study was conducted at a tertiary cancer center with a relatively small sample size, which limits generalizability. Nevertheless, we believe that our findings offer important preliminary insights and may inform future research and policy in similar settings.

In conclusion, we identified that a significant proportion of Mexican patients with advanced cancer present to the ED with diverse symptoms related to cancer and its treatment and that more than half of these visits are potentially avoidable. Our results underscore the urgent need to strengthen outpatient and palliative care services for patients with advanced cancer in Mexico. While larger multicenter studies are needed to incorporate patient-reported outcomes and cost-effectiveness analyses, we propose feasible actions such as routine palliative assessments during oncology follow-up and the development of clinical pathways for the outpatient management of common symptoms.

## Conflicts of interest

Yanin Chávarri-Guerra declares research support from Roche and Pfizer; Speakers bureau from Gilead, Astra Zeneca, Lilly and MSD, Novartis, Pfizer, Gilead and Lilly travel expenses; Astra Zeneca Advisory role.

## Funding

This research received no specific grant from any funding agency in the public, commercial or not-for-profit sectors.

## Figures and Tables

**Table 1.** Demographic and clinic characteristics.

Characteristics	*n* = 77 (%)
Mean age (range)	62 (47–77)
Gender (male)	42 (55)
Educational level	
None	5 (7)
Elementary school	10 (13)
Middle school and High school	39 (50)
Bachelor’s degree	23 (30)
Health insurance	
No health insurance	30 (40)
Insured	47 (60)
Employment status	
Formally employed	26 (34)
Self-employed	21 (27)
Student	2 (3)
Retired	15 (19)
Unemployed	13 (17)
Oncological diagnosis	
New diagnosis	68 (88)
Recurrence	9 (12)
Location of the primary tumor	
Gastrointestinal	39 (53)
Genitourinary	16 (21)
Other	22 (26)
ECOG performance status	
0	3 (4)
1	23 (30)
2	14 (18)
3 o 4	7 (9)
Not reported	30 (39)
Treatment received	
Chemotherapy	46 (60)
Other	9 (12)
No treatment	22 (28)

ECOG Performance Status: Eastern Cooperative Oncology Group Performance Status

**Table 2.** Reasons for ED visits.

	Total *n* = 96 (%)	Avoidable *n* = 61 (%)
Infection	18 (18.7)	5 (8.1)
GI disorders	21 (21.8)	14 (22.9)
Pain	15 (15.6)	15 (24.5)
Ascites	14 (14.5)	14 (22.9)
Anemia	4 (4.1)	4 (6.5)
Catheter dysfunction	4 (4.1)	3 (4.9)
Dyspnea	2 (2.0)	0
Other	18 (18.7)	6 (9.8)

GI: Gastrointestinal
